# *Caenorhabditis elegans* germ granules accumulate hundreds of low translation mRNAs with no systematic preference for germ cell fate regulators

**DOI:** 10.1242/dev.202575

**Published:** 2024-07-10

**Authors:** Alyshia Scholl, Yihong Liu, Geraldine Seydoux

**Affiliations:** HHMI and Dept. of Molecular Biology and Genetics, Johns Hopkins University School of Medicine, Baltimore, MD 21205, USA

**Keywords:** Germ plasm, Germ granules, Nanos, MEG-3, Germline, *C*. *elegans*

## Abstract

In animals with germ plasm, embryonic germline precursors inherit germ granules, condensates proposed to regulate mRNAs coding for germ cell fate determinants. In *Caenorhabditis elegans*, mRNAs are recruited to germ granules by MEG-3, a sequence non-specific RNA-binding protein that forms stabilizing interfacial clusters on germ granules. Using fluorescence *in situ* hybridization, we confirmed that 441 MEG-3-bound transcripts are distributed in a pattern consistent with enrichment in germ granules. Thirteen are related to transcripts reported in germ granules in *Drosophila* or *Nasonia*. The majority, however, are low-translation maternal transcripts required for embryogenesis that are not maintained preferentially in the nascent germline. Granule enrichment raises the concentration of certain transcripts in germ plasm but is not essential to regulate mRNA translation or stability. Our findings suggest that only a minority of germ granule-associated transcripts contribute to germ cell fate in *C. elegans* and that the vast majority function as non-specific scaffolds for MEG-3.

## Teaser

**Highlighted Article:** Germ granule-enriched RNA provides a structural, not functional, role in germ cell fate specification in *C. elegans*.

## INTRODUCTION

In animals, somatic and germ lineages separate during embryogenesis when a small number of cells become specified as primordial germ cells (PGCs), the precursors to the germline. In several species, including model systems such as *Caenorhabditis elegans*, *Drosophila*, *Xenopus* and zebrafish, PGCs inherit ‘germ plasm’, a specialized cytoplasm that contains germ granules, condensates of maternal mRNAs and proteins (Chiappetta et al., 2022). Irradiation of germ plasm in the frog *Rana pipiens* eliminates PGCs (Smith, 1966) and transplantation of germ plasm in *Drosophila* and *Xenopus* results in ectopic PGCs (Illmensee and Mahowald, 1974; Tada et al., 2012). Together with the observation that ribosomes accumulate at the periphery of germ granules in *Drosophila* (Mahowald, 1968), these findings led to the hypothesis that germ granules deliver to PGCs specific maternal mRNAs coding for germ cell fate determinants. Consistent with this view, in animals with germ plasm, the germ cell fate regulator Nanos is encoded by a maternal mRNA that is enriched in germ granules (Chiappetta et al., 2022).

The complete set of mRNAs associated with germ granules has not been determined in any organism. Depending on the species, a handful to dozens of germ granule transcripts have been reported, but their role in germ cell fate is not always clear. For example, an *in situ* hybridization survey in *Drosophila* identified 58 transcripts that exhibit a distribution consistent with localization to germ granules (Rangan et al., 2009), but only four (*Nos*, *CycB*, *pgc* and *gcl*) have been implicated in the formation or fate of PGCs (Trcek and Lehmann, 2019). A survey in the wasp *Nasonia* identified 26 transcripts enriched in the oosome, but only three are homologous to *Drosophila* germ granule transcripts, including *nanos* (Quan et al., 2019). In contrast to mRNAs, proteins in germ granules are mostly conserved, including Argonautes, the VASA family of RNA-dependent ATPases, and regulators of RNA translation and decay (Cassani and Seydoux, 2023; Chiappetta et al., 2022).

In this study, we conducted a large-scale *in situ* hybridization screen to identify mRNAs that are enriched in granules in the germ plasm of *C. elegans*. The *C. elegans* germ plasm assembles in the zygote posterior and segregates asymmetrically to four successive germline blastomeres (P_1_ to P_4_). The germline founder P_4_ divides symmetrically to give rise to the PGCs, Z2 and Z3 (Fig. S1A). At least two condensate types segregate in germ plasm to the PGCs: P granules and germline P-bodies. P granules are liquid-like condensates present throughout the life cycle of germ cells that contain Argonautes and the phase-separating PGL proteins (Batista et al., 2008; Wang and Reinke, 2008). Germline P-bodies are a complex collection of condensates that contain regulators of RNA translation and decay (Cassani and Seydoux, 2022). P granules and germline P-bodies form multi-condensate assemblies (‘germ granules’) that segregate together in germ plasm (Cassani and Seydoux, 2022). These assemblies are stabilized in germ plasm by four, partially redundant, intrinsically disordered proteins (MEG-1 to MEG-4) that are enriched specifically in germ plasm. Best understood are MEG-3 and MEG-4, which form interfacial, low-dynamic assemblies that function as Pickering agents that stabilize P granules (and, redundantly with MEG-1/2, germline P-bodies) in germ plasm to ensure their segregation to PGCs (Wang et al., 2014; Putnam et al., 2019; Folkmann et al., 2021).

Several lines of evidence indicate that MEG-3 and MEG-4 are responsible for recruiting the bulk of mRNAs to germ granules. In wild-type embryos, *in situ* hybridization using an oligo(dT) probe to detect poly(A)^+^ mRNAs, or a probe against the Nanos homolog *nos-2*, reveal nearly micron-sized granules in P blastomeres and Z2 and Z3 (Seydoux and Fire, 1994; Subramaniam and Seydoux, 1999). In *meg-3 meg-4* embryos, mRNAs distribute uniformly throughout the cytoplasm of germline blastomeres, as in somatic blastomeres (Lee et al., 2020). PGL condensates and germline P-bodies assemble in early *meg-3 meg-4* zygotes, but are not preferentially stabilized in germ plasm and are cleared by the four-cell stage (PGL condensates) or still segregate to PGCs but as smaller condensates (germline P-bodies) (Cassani and Seydoux, 2022; Wang et al., 2014). MEG-3 is a sequence non-specific RNA-binding protein that uses a long, N-terminal, intrinsically disordered domain to bind RNA and a C-terminal motif to bind PGL proteins (Schmidt et al., 2021). Immunoprecipitation from embryonic lysates of MEG-3::GFP cross-linked to RNA identified ∼500 transcripts (Lee et al., 2020). MEG-3 crosslinks show no sequence specificity and tend to favor transcripts with low ribosome coverage (Lee et al., 2020). Eighteen MEG-3-bound mRNAs were validated by *in situ* hybridization as germ granule-enriched, including the Nanos homolog *nos-2* and the TRIM32 homolog *grif-1*, which are translationally activated in the germline founder cell, maintained into Z2 and Z3, and required redundantly for germ cell fate (Lee et al., 2020). In the present study, we examined by *in situ* hybridization an additional 469 MEG-3-bound transcripts for a total of 487 mRNAs and verified that 90% are enriched in a granular pattern in P blastomeres. Remarkably, unlike *nos-2* and *grif-1*, most are not maintained as germline-enriched and do not appear to encode factors with germline-specific functions. Together with prior findings (Folkmann et al., 2021; Lee et al., 2020; Parker et al., 2020; Schmidt et al., 2021), our observations suggest that most mRNAs are recruited to germ granules not for regulation, but to enhance MEG-3 condensation and stabilize protein-rich condensates in germ granules.

## RESULTS

### The majority of MEG-3-bound transcripts are enriched in foci in P blastomeres but not in PGCs

**Fig. 1.**
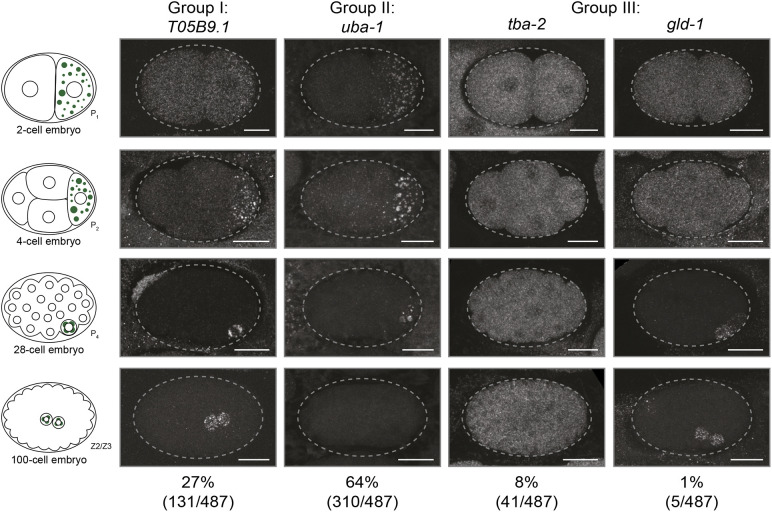
**Most MEG-3-bound transcripts concentrate in foci in germline blastomeres but are not maintained in PGCs.** Photomicrographs of wild-type embryos staged as indicated in the diagrams and hybridized to the gene-specific smFISH probes indicated at the top. Percentages (bottom) indicate the proportion of transcripts with a similar smFISH pattern out of 487 total transcripts analyzed (see Materials and Methods). Dashed lines indicate embryo boundaries. Scale bars: 10 µm. Representative photomicrographs for the 487 transcripts included in the screen are accessible from the Dryad Digital Repository (Scholl, 2024): dryad.02v6wwq98 and tabulated in Table S3.

Using probes against 487 MEG-3-bound transcripts identified in Lee et al. (2020), we performed single-molecule fluorescence *in situ* hybridization (smFISH) in fixed embryos (two-cell to 100-cell stage). In this developmental period, somatic blastomeres turn over most maternal mRNAs and activate zygotic transcription, whereas P blastomeres maintain maternal mRNAs and do not activate zygotic transcription of mRNAs until after the division of P_4_ into Z2 and Z3 (Seydoux and Dunn, 1997; Seydoux and Fire, 1994). Consistent with localization to germ granules, most transcripts surveyed (441/487; 91%) were concentrated in foci in at least one P blastomere. All transcripts, however, could also be detected throughout the cytoplasm, indicating that no transcript uniquely localized to germ granules (Fig. 1). As expected, we identified many transcripts that, like *nos-2* and *grif-1*, localized to two cells in the 100-cell stage, consistent with maintenance in Z2 and Z3. We designate these mRNAs as Group I (131 out of 441 foci-enriched transcripts, 30%). Surprisingly, however, the majority of foci-enriched transcripts were either not detected in the 100-cell stage or did not localize in a pattern consistent with Z2 and Z3. We designate these mRNAs as Group II (310/441, 70%). Group III corresponds to the minority of transcripts that did not concentrate in foci in any P blastomeres (Group III, 46/487 of MEG-3 bound mRNAs, 9%). Interestingly, five Group III transcripts exhibited a pattern consistent with maintenance in Z2 and Z3 (Fig. 1). These observations suggest that localization to germ granules is neither essential nor sufficient for maternal transcripts to be maintained into PGCs.

All three groups contained ∼50% genes annotated in WormBase (Davis et al., 2022) as exhibiting an ‘embryonic lethal’ phenotype (Fig. S1B). We used gene ontology (GO) term analysis to identify functional classes over-represented in Group I, II or III mRNAs compared with GO terms over-represented in the early embryo transcriptome (Fig. S1C). We found that Group I was enriched for GO terms related to germline development and reproduction, whereas Group II was enriched for GO terms related to metabolic processes. In contrast, Group III was enriched for GO terms related to embryonic development, as also observed for the whole embryo transcriptome. Consistent with prior studies using individual-nucleotide resolution UV crosslinking and immunoprecipitation (iCLIP) suggesting that low translation drives mRNAs into germ granules (Lee et al., 2020), Group I and II mRNAs had lower ribosome occupancy on average compared with Group III (Fig. S1D). Group III mRNAs, in contrast, exhibited more ribosome footprints on average (normalized for RNA abundance) and were also more abundant on average compared with Group I and II mRNAs (Fig. S1E), raising the possibility that Group III transcripts arose as contaminants in the MEG-3 iCLIP experiments of Lee et al. (2020).

We conclude that most MEG-3-bound mRNAs identified in iCLIP experiments are granule-enriched transcripts, with only ∼10% corresponding to false positives. Surprisingly, however, most transcripts (70%, Group II) are not enriched in PGCs and do not appear to code for factors with germline-specific functions.

### Only a minority of Group I mRNAs contribute to specification of germ cell fate

**Fig. 2.**
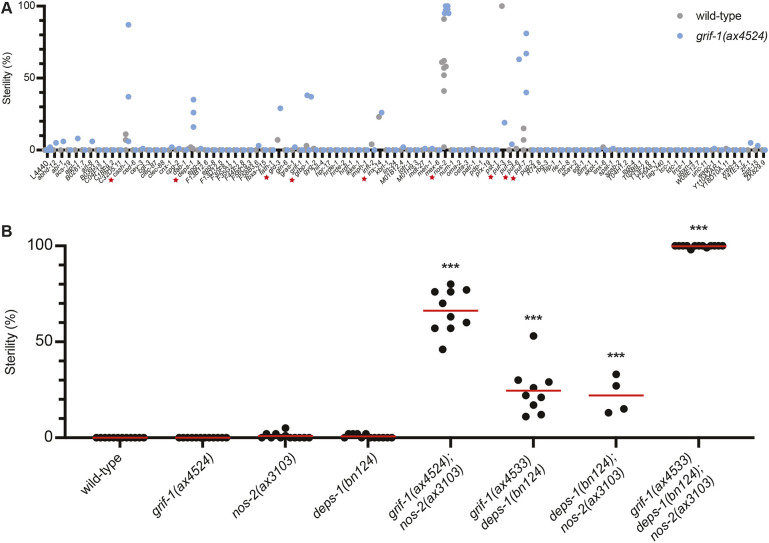
**A minority of Group I transcripts contribute to germ cell fate as assayed by RNA-mediated interference.** (A) Percentage sterility (*y*-axis) of broods (>30 self-progeny) derived from 10-20 wild-type (gray dots) or *grif-1(ax4524)* (blue dots) mothers exposed to RNAi by feeding against the genes indicated in the *x*-axis. Red stars indicate RNAi treatments that yielded a significant percentage of sterile progeny (Fisher's exact test, *P*<0.01). *grif-1(ax4524)* is a full deletion of the *grif-1* locus (100% viable and fertile; Fig. S2E,F). For *cash-1*, *deps-1*, *gtbp-1* and *puf-7 n*=3, L4440 and *nos-2 n*=8 biological replicates. For all other genes, *n*=1 biological replicate. (B) Graph showing the percentage sterility (*y*-axis) of the genotypes as listed on the *x*-axis. Each dot (biological replicate) corresponds to a brood (>30 self-progeny) from 10-20 mothers, except for *grif-1 deps-1; nos-2* worms, where dots correspond to broods (>30 self-progeny) derived from a single mother of the indicated genotype. Red lines indicate the mean. Refer to Fig. S2B for crosses used to generate triple mutant. Data for the triple mutant are also shown in Fig. S2C. ***P≤0.0001 (unpaired *t*-test compared with wild type). In contrast to *nos-2(RNAi)* (A), *nos-2(ax3103)* displays very low sterility on its own, because RNAi culture conditions enhance the *nos-2* sterility phenotype (Fig. S2D). For wild-type, *grif-1(ax4524)*, *nos-2(ax3103)*, *deps-1(bn124) n*=12, *grif-1(ax4533) deps-1(bn124) n*=9, *deps-1(bn124); nos-2(ax3103) n*=4, *grif-1(ax4533) deps-1(bn124); nos-2(ax3103) n*=13 biological replicates.

Group I genes included two mRNAs, *nos-2* and *grif-1*, required redundantly for germ cell fate (Lee et al., 2020). *nos-2* is a Nanos homolog that promotes the turnover of maternal mRNAs in Z2 and Z3 (Lee et al., 2017) and *grif-1* codes for a predicted E3 ubiquitin ligase that promotes the turnover of RNA regulators in Z2 and Z3 (Oyewale and Eckmann, 2022). *grif-1* and *nos-2* single mutants generate mostly fertile progeny, whereas *grif-1; nos-2* double mutants give rise to ∼65% of progeny with no germline (‘white steriles’) (Fig. 2B; Lee et al., 2020). To investigate whether other Group I genes contribute to germline formation, we inactivated by RNA interference (RNAi) 127 Group I mRNAs in wild-type and *grif-1(ax4524)* adult hermaphrodites and scored their progeny for white steriles. Of the 127 genes tested, 22% (28/127) resulted in dead embryos or larvae. Among the remainder, only nine genes (7%) gave rise to ‘white sterile’ progeny in wild-type hermaphrodites (*puf-3*), in *grif-1* hermaphrodites (*cash-1*, *deps-1*, *gld-3*, *gtbp-1*, *puf-6* and *puf-7*) or in both (*inx-14* and *nos-2*; Fig. 2A). All have been implicated previously in various aspects of germline development (Eckmann et al., 2004; Haupt et al., 2020; Lu et al., 2022; Spike et al., 2008; Starich et al., 2014; Subramaniam and Seydoux, 1999).

Among this group, the distribution of *deps-1* transcript was most similar to that of *grif-1* and *nos-2*, with robust *deps-1* transcript levels maintained in the germ lineage to the 100-cell stage (Fig. S2A). DEPS-1 codes for a protein that localizes to germ granules and has been implicated in piRNA-dependent gene silencing (Spike et al., 2008; Suen et al., 2020). To explore a possible role for *deps-1* in germ cell fate specification in parallel to *grif-1* and *nos-2*, we generated double and triple mutant combinations. We found that, whereas *deps-1* single mutants are fertile at 20°C, *grif-1 deps-1* and *deps-1; nos-2* double mutants give rise to ∼25% white sterile progeny, similar to *grif-1; nos-2* double mutants (Fig. 2B). Remarkably, the triple mutant *grif-1 deps-1; nos-2* produced 100% white sterile progeny, consistent with each gene contributing in parallel to germ cell fate specification (Fig. 2B; Fig. S2B,C). We conclude that a minority of Group I genes encode maternal transcripts that contribute to establishment of the germline in embryos, including *deps-1*, *nos-2* and *grif-1*. The vast majority, however, do not appear to be essential for initiation of germ cell development under standard laboratory growth conditions (20°C), although we cannot exclude the possibility that our RNAi treatments were ineffective against certain genes or that certain genes have germline functions that do not result in a no-germline ‘white sterile’ phenotype.

### MEG-3-bound mRNAs are enriched at variable levels in P_4_

**Fig. 3.**
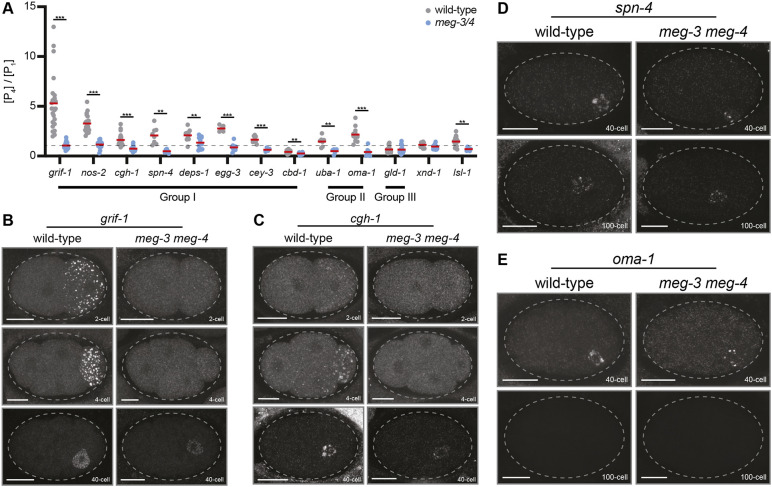
**MEG-3-bound mRNAs accumulate at variable levels in the germline founder cell P_4_.** (A) The relative concentration in P_4_ compared with P_1_ (*y*-axis) for the transcripts listed (*x*-axis) as determined by smFISH. Each dot corresponds to a single P_4_ cell normalized to the average of at least three P_1_ cells. Red lines indicate the mean. Dashed line indicates 1. ***P≤0.0001, **P≤0.001 (unpaired *t*-test comparing wild type and *meg-3 meg-4*). Refer to B,C for example photomicrographs of P_1_ (two-cell) and P_4_ (40-cell). For wild-type data: *grif-1 n*=25, *nos-2 n*=20, *cgh-1 n*=20, *spn-4 n*=8, *deps-1 n*=13, *egg-3 n*=5, *cey-3 n*=9, *cbd-1 n*=18, *uba-1 n*=7, *oma-1 n*=12, *gld-1 n*=25, *xnd-1 n*=12, *lsl-1 n*=19 biological replicates. For *meg-3/4* data: *grif-1 n*=22, *nos-2 n*=15, *cgh-1 n*=13, *spn-4 n*=6, *deps-1 n*=13, *egg-3 n*=10, *cey-3 n*=4, *cbd-1 n*=21, *uba-1 n*=6, *oma-1 n*=8, *gld-1 n*=21, *xnd-1 n*=11, *lsl-1 n*=6 biological replicates. (B-E) Representative photomicrographs of embryos of the indicated genotype and stage hybridized to gene-specific smFISH probes as indicated. Scale bars: 10 µm. B and C show *meg-3 meg-4*-dependent enrichment of transcripts in granules and P_4_. D and E show *meg-3 meg-4*-independent maintenance (D) and turnover (E) of transcripts from P_4_ (40-cell) to Z2 and Z3 (100-cell). Note that in all cases, transcripts are cleared from somatic cells by the 40-cell stage. Fig. S3C,D shows additional examples.

P blastomere divisions generate successively smaller P blastomeres that inherit most germ granules (Fig. S1A; Deppe et al., 1978; Strome and Wood, 1982). mRNAs that are enriched in germ granules therefore are expected to become more concentrated in P blastomeres with each division. To explore this prediction, we quantified mRNA concentration (intensity of smFISH signal/cell area, see Materials and Methods) in P_4_ (newly born, 28- to 50-cell stage) versus P_1_ (two-cell stage) for ten MEG-3-bound mRNAs that are enriched in foci in P blastomeres (Fig. 3A-C). If 100% of a specific transcript localized to germ granules, each asymmetric division should increase the concentration of that transcript by approximately twofold, resulting in an eightfold increase in concentration between P_1_ and P_4_ (three divisions). Given that all mRNAs localized to both granules and cytoplasm, we expected lower levels of enrichment. As expected, nine out of the ten mRNAs scored above 1 (more concentrated in P_4_ compared with P_1_), with modest enrichment (approximately twofold) for most mRNAs, except for *grif-1* (fivefold enrichment; Fig. 3A-C). Localization to foci and P_4_ enrichment was dependent on *meg-3* and *meg-4* (Fig. 3A-C). In *meg-3 meg-4* mutants, mRNAs were maintained at similar levels between P_1_ and P_4_, unlike in somatic blastomeres where all mRNAs tested were degraded by the ∼40-cell stage (Fig. 3A-C). We conclude that localization to granules in P blastomeres can lead to enrichment of transcripts in P_4_, but to a variable degree with some transcripts barely enriched. These observations also confirm that maternal mRNAs do not require granule localization to remain stable in P_1_ through P_3_ (Lee et al., 2020).

We also examined two transcripts that, like *grif-1* and *nos-2*, are translationally activated in P_4_ and code for factors that contribute to germ cell fate (Mainpal et al., 2015; Rodriguez-Crespo et al., 2022). *xnd-1* and *lsl-1* are low-abundance maternal transcripts that scored above control in only one of two MEG-3 iCLIP replicates and thus were not retained as bona fide MEG-3-bound transcripts in the original study of Lee et al. (2020). We detected a weak *meg-3 meg-4*-dependent enrichment in P_4_ for *lsl-1* (1.4-fold), but not for *xnd-1* (Fig. 3A; Fig. S3A). We conclude that robust enrichment in P_4_, best exemplified by *grif-1*, is not a conserved feature of all mRNAs that localize to germ granules or that code for germ cell fate determinants.

### Enrichment in granules is not essential for RNA regulation in P blastomeres

We previously reported that translational repression and activation proceeds as in wild type in *meg-3 meg-4* embryos for *grif-1*, *nos-2* and *xnd-1* (Cassani and Seydoux, 2022; Lee et al., 2020). We confirmed that *lsl-1* is also properly regulated in *meg-3 meg-4* mutants (Fig. S3B; Rodriguez-Crespo et al., 2022). To determine whether mRNA maintenance or turnover in P_4_ requires localization to germ granules, we examined two Group I and two Group II mRNAs in 40-cell and 100-cell *meg-3 meg-4* embryos. We found that Group I mRNAs were maintained (Fig. 3D; Fig. S3C) and Group II mRNAs were turned over in *meg-3 meg-4* mutants, as in wild type (Fig. 3E; Fig. S3D). We conclude that enrichment in germ granules is not essential for regulation of mRNA stability, turnover or translation. Enrichment in germ granules, however, can increase the concentration of some transcripts, such as *nos-2* and *grif-1*, in the germline founder cell above their initial concentration in the zygote.

### Enrichment in germ granules correlates with low ribosome occupancy

**Fig. 4.**
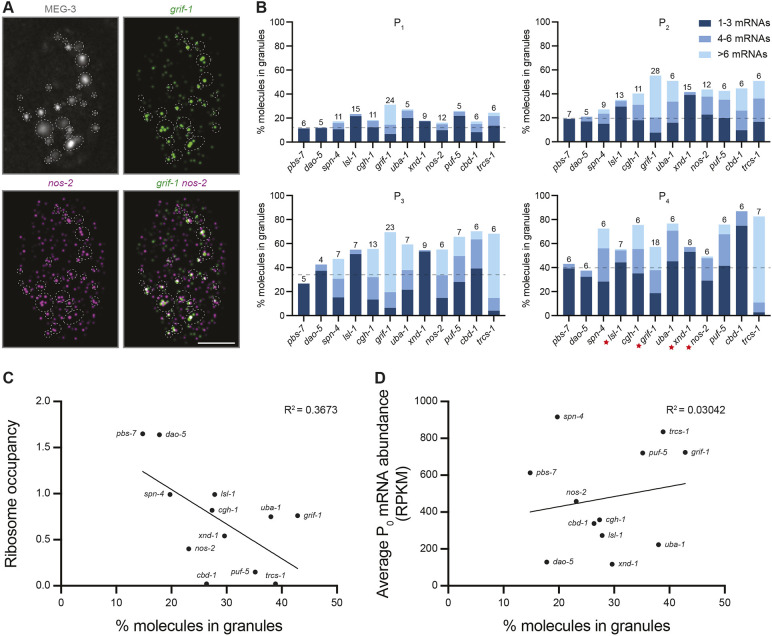
**Airlocalize analysis of RNA enrichment and cluster formation in germ granules.** (A) Representative Airlocalize-processed, single-slice image of a P_2_ blastomere expressing MEG-3::GFP and probed for *nos-2* and *grif-1* RNAs. Scale bar: 5 µm. Note that the *in situ* conditions do not preserve individual MEG-3 condensates; dashed lines indicate MEG-3-positive areas, which correspond to groupings of multiple condensates, including P granules and germline P-bodies. Brightness of images shown here was enhanced for clarity, resulting in artifactual instances of transcript colocalization; see Fig. 5A for a higher magnification, non-enhanced example (Materials and Methods). (B) Bar graphs showing the mean percentage of molecules colocalized with MEG-3::GFP granules (*y*-axis) for each P blastomere and transcript (*x*-axis). Values at the top of each bar indicate the number of P blastomeres examined (data for individual blastomeres are shown in Fig. S4D). Transcripts are ordered based on their average ribosome occupancy (high to low; values shown in C). Note that the volume occupied by germ granules increases with each P blastomere (Fig. S4E) and hence the percentage of mRNA molecules in germ granules also increases even for the well-translated transcripts *pbs-7* and *dao-5* (which are not enriched, but also not excluded, from the granules). Values above the dashed line (average of *pbs-7* and *dao-5*) denote enrichment in granules above the cytoplasm. Colors inside each bar indicate the percentage of molecules in granules colocalized in ‘clusters’ containing multiple mRNA molecules as indicated in the key. Red stars indicate transcripts that become translationally activated in P_4_. (C,D) Graphs comparing ribosome occupancy (*y*-axis; data from Lee et al., 2020) or average RPKM (reads per kilobase per million mapped reads) in P_0_ (*y*-axis; data from Tintori et al., 2016) with the percentage of molecules in germ granules (*x*-axis; average across P_1_ to P_3_ stages). The best-fit line is a simple linear regression.

To quantify RNA localization in germ granules, we repeated smFISH experiments in a strain expressing MEG-3::GFP (tagged at the endogenous locus; Fig. S4A). Because our smFISH protocol does not preserve the interfacial distribution of MEG-3::GFP in germ granules (Folkmann et al., 2021), we could only locate RNAs relative to germ granules generically, not specific condensate types within the granules. We used the image analysis software Airlocalize (Lionnet et al., 2011; Trcek et al., 2015) to determine the position and brightness of smFISH signals (Materials and Methods). For each transcript, smFISH signals were calibrated under the assumption that the majority of smFISH intensities in somatic blastomeres correspond to single transcripts (Fig. S4B). P blastomeres exhibited higher smFISH signal intensities compared with somatic blastomeres, including some corresponding to four or more mRNA molecules in one diffraction-limited spot (∼150 nm; Fig. S4C). We refer to these as ‘clusters’. Using *z*-stack images spanning whole P blastomeres, we calculated the percentage of RNA molecules in MEG-3-positive germ granules and the percentage coalesced in clusters (Fig. 4B; Fig. S4D).

**Fig. 5.**
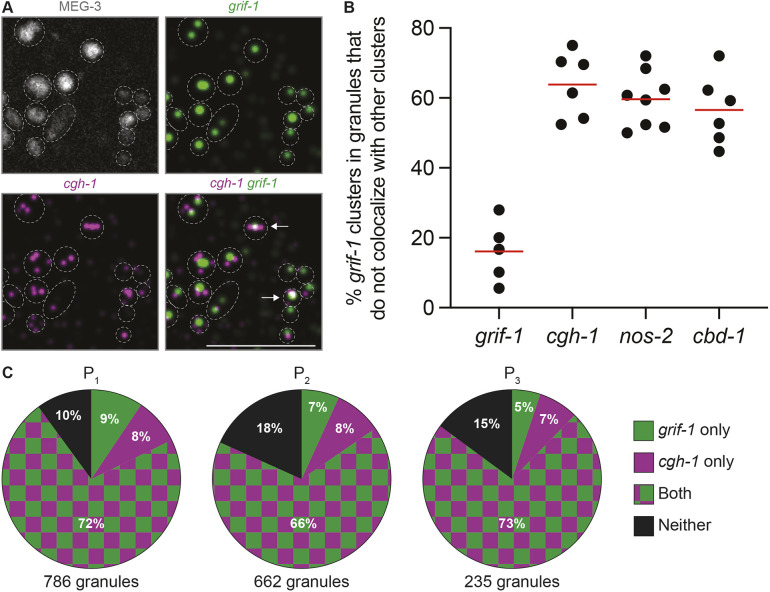
**Most *grif-1* clusters do not overlap with *cgh-1*, *nos-2* or *cbd-1* clusters.** (A) Representative Airlocalize-processed single-slice images of a P_2_ blastomere expressing MEG-3::GFP and probed for *cgh-1* and *grif-1* RNAs. Dashed lines outline MEG-3 granules. In the merged image, arrows indicate rare instances of colocalization between two RNA clusters. Scale bar: 5 µm. (B) Percentage of *grif-1* clusters in germ granules of P_2_ blastomeres (*y*-axis) that do not colocalize with other RNA clusters (*x*-axis). Each dot represents a single P_2_ blastomere. (C) Pie charts showing the percentage of MEG-3-positive granules containing *grif-1* transcripts (green), *cgh-1* transcripts (purple), both (checkered) or neither (black). The number of MEG-3-positive granules analyzed is listed below each chart. Note that each MEG-3-positive granule corresponds to a grouping of multiple condensates stabilized by MEG proteins, including P granules and germline P-bodies (Cassani and Seydoux, 2022).

We examined 12 transcripts, including four transcripts known to code for germ cell fate determinants translated in P_4_ (*nos-2*, *grif-1*, *xnd-1* and *lsl-1*), six germ granule-enriched transcripts selected to represent a range of abundance and ribosome coverage, and two non-MEG-3 bound controls. *pbs-7* and *dao-5* are two well-translated transcripts that do not bind MEG-3 or enrich in germ granules, unless translation is globally inhibited (Lee et al., 2020)*. pbs-7* and *dao-5* are not excluded from germ granules and thus serve as a baseline to control for the increasing volume occupied by germ granules from P_1_ to P_4_ (Fig. S4E). As expected, all transcripts tested were enriched in granules above the base level of *pbs-7* and *dao-5* (dashed line in Fig. 4B; see Fig. S4D for statistics), but to varying degrees and with no transcript localizing exclusively to granules at any stage. *grif-1* was the most highly enriched transcript in all P blastomeres except P_4_, where the percentage of *grif-1* molecules in germ granules dropped closer to levels observed for *pbs-7* and *dao-5* at that stage (Fig. 4B; Fig. S4D). A similar drop was observed for *lsl-1*, *xnd-1* and *nos-2*, which, like *grif-1*, become translated in P_4_ (red stars in Fig. 4B). This change was not due to RNA degradation in the granules because the concentration of these transcripts increased from P_3_ to P_4_ (Fig. S4F). These observations confirm that MEG-3-bound transcripts, including low abundance *xnd-1* and *lsl-1*, are enriched in MEG-3-marked germ granules and suggest that this enrichment is reversible and anti-correlated with translation.

To explore further the correlation between granule enrichment and translational status, we compared the percentage of molecules in granules (average across P_1_ to P_3_) with average ribosome coverage (as determined by ribosome foot-printing analysis in mixed-stage early embryos; Lee et al., 2020) and with mRNA abundance (as determined by RNA sequencing of zygotes; Tintori et al., 2016). We observed a negative correlation with ribosome coverage (Fig. 4C) and no correlation with mRNA abundance (Fig. 4D), confirming that germ granules specifically favor low-translation transcripts, as previously suggested by Lee et al. (2020). We note, however, that the correlation is relatively weak (R^2^=0.37) with *grif-1* accumulating in granules to a greater extent than predicted from ribosome occupancy.

### Transcripts in germ granules become concentrated in sub-granule clusters

In the granules, mRNAs did not distribute uniformly but rather accumulated in sub-granular clusters containing multiple colocalized molecules. The number of molecules in clusters varied between transcripts (Fig. 4B). *grif-1* formed the most concentrated clusters, accumulating as many as 35 mRNA molecules in a single cluster (Fig. S5). *nos-2* also formed relatively concentrated clusters, accumulating as many as 19 molecules (Fig. S5; consistent with values reported by Parker et al., 2020). The two other transcripts coding for germ cell fate determinants, *xnd-1* and *lsl-1*, however, formed very few clusters (Fig. 4B).

In *Drosophila*, clusters containing different mRNA species do not overlap in germ granules (‘homotypic clusters’; Eagle et al., 2018; Little et al., 2015; Niepielko et al., 2018; Trcek et al., 2015, 2020). Similar observations have been reported for two germ granule-enriched mRNAs in *C. elegans* (Parker et al., 2020). Consistent with these observations, in two-color smFISH experiments, ∼60% of *grif-1* clusters did not overlap with *cgh-1*, *nos-2* or *cbd-1* clusters (Fig. 5A,B), even though they were often found in the same MEG-3-positive granule (Fig. 5C). We note, however, that because each MEG-3-positive granule corresponds to a grouping of germline P-bodies and P granules, it is possible that homotypic RNA clusters reside in separate condensates.

### Clustering is enhanced by MEG-3/4

**Fig. 6.**
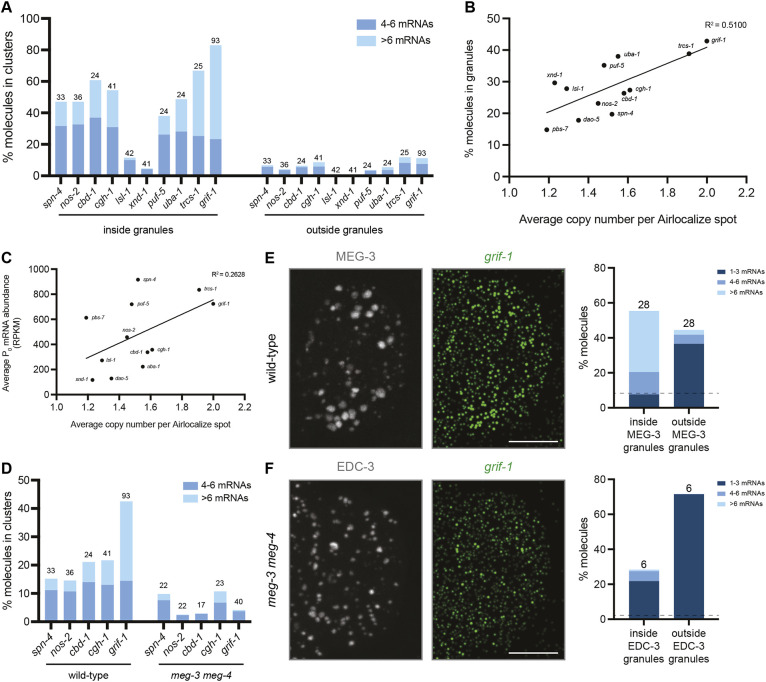
**mRNA copy number per Airlocalize focus correlates with mRNA abundance and localization to germ granules.** (A) Percentage of RNA molecules in clusters (*y*-axis) in wild-type embryos comparing RNAs inside germ granules (marked with MEG-3::GFP) versus RNAs outside germ granules. Values at the top of each bar indicate the number of P blastomeres examined for each transcript (all P cell stages combined). Genes are ordered from low to high average percentage of molecules in granules (see B). (B,C) Graphs comparing the percentage of molecules in granules (B; P_1_ to P_3_ data from Fig. 4) or average RPKM in P_0_ zygotes (C; *y*-axis; data from Tintori et al., 2016) with the average mRNA copy number per Airlocalize spot (*x*-axis; P_1_ to P_3_ stages). The best-fit line is a simple linear regression. (D) Percentage of all RNA molecules in clusters (*y*-axis) in wild-type and *meg-3 meg-4* embryos (all P cells combined; wild-type values are same as shown in A but considering all molecules whether inside or outside granules). Values at the top of each bar indicate the number of P blastomeres examined. (E,F) Representative Airlocalize-processed maximum-projection images of P_2_ blastomeres probed for *grif-1* RNA and expressing MEG-3::GFP (wild type; E) or mNG::EDC-3 (*meg-3 meg-4* mutant; F). The graphs show the percentage of *grif-1* molecules (*y*-axis) inside and outside MEG-3::GFP granules in wild-type P_2_ blastomeres (E), and inside and outside mNG::EDC-3 granules in *meg-3 meg-4* P_2_ blastomeres (F). Colors inside each bar indicate the percentage of molecules colocalized in clusters containing multiple mRNA molecules as indicated in the key. Dashed lines represent the percentage of the cytoplasm occupied by MEG-3 or EDC-3 granules (Fig. S4E). Values at the top of each bar indicate the number of P_2_ blastomeres examined. Scale bars: 10 µm.

Most clusters localized to MEG-3-positive granules with only a minority detected in the cytoplasm away from granules, suggesting that clustering is enhanced by MEG-3 in the granule environment (Fig. 6A). Consistent with this hypothesis, for the set of 12 transcripts, clustering efficiency correlated with granule enrichment (Fig. 6B) more than with mRNA abundance (Fig. 6C). Neither correlation, however, was absolute and both factors likely contribute. For example, despite similar enrichment in granules, the low-abundance transcripts *xnd-1* and *lsl-1* formed low copy number clusters compared with the relatively more abundant *nos-2*. These observations suggest that clustering efficiency is influenced by MEG-3, mRNA abundance, and likely other unknown factors.

To examine directly the dependence of clustering on MEG-3/4, we repeated the Airlocalize analysis in *meg-3 meg-4* mutants for five of the most efficient cluster-forming mRNAs. We observed clusters in *meg-3 meg-4* embryos, but at lower frequencies compared with wild type (Fig. 6D). *meg-3 meg-4* embryos do not maintain P granules but do maintain germline P-bodies in germ plasm (Cassani and Seydoux, 2022). We found that *grif-1* molecules became concentrated in germline P-bodies in *meg-3 meg-4* embryos but less efficiently and in fewer clusters compared with wild type (Fig. 6E,F). We conclude that MEG-3/4 is essential for efficient RNA recruitment and clustering in germ granules.

## DISCUSSION

We characterized by fluorescence *in situ* hybridization 487 mRNAs recovered by Lee et al. (2020) in iCLIP experiments with MEG-3, the RNA-binding protein responsible for recruiting mRNAs to germ granules in *C. elegans* embryos. Our findings lead to two main conclusions. First, the majority of MEG-3-bound mRNAs are enriched in foci in P blastomeres, consistent with recruitment to germ granules by MEG-3, but this localization does not appear to be predictive of a role in germline development. Most MEG-3-bound transcripts are not enriched in PGCs and only a small percentage of those that persist in PGCs contribute to germ cell fate as assayed by RNAi. Second, low-translation mRNAs are preferentially recruited into germ granules, but do not require granule localization for translational repression, activation, or mRNA maintenance and/or turnover. Granule localization boosts the concentration of maternal transcripts in the germline founder cell, but to different degrees dependent on the transcript. As first reported in *Drosophila* (Little et al., 2015; Niepielko et al., 2018; Trcek et al., 2015, 2020), we find that mRNAs in germ granules coalesce into spatially segregated clusters containing several copies of the same transcript and that strong clustering is dependent on MEG-3. Together, these observations suggest that MEG-3 recruits to germ granules a broad range of translationally repressed ribonucleoprotein complexes that self-associate in the granules, but this recruitment is not systematically selective for transcripts with germline-specific functions and is not essential for mRNA regulation. These observations have implications for germ granule assembly and the role of mRNAs in germ granules, which we discuss below.

### MEG-3 recruits low-translation mRNAs to germ granules

Our systematic *in situ* hybridization survey identified 441 MEG-3-bound transcripts that are enriched in granules in P blastomeres, consistent with enrichment in germ granules. Because our smFISH protocol does not preserve the integrity of individual condensates in germ granules, we do not know whether MEG-3-bound transcripts are recruited specifically to MEG-3 assemblies that associate with P granules, germline P-bodies or both. Approximately 50% correspond to genes required generally for embryonic viability and 70% produce transcripts that are not enriched in PGCs. Several of our observations are consistent with MEG-3 binding to translationally repressed transcripts with no sequence preference, as first suggested by Lee at al. (2020) who showed that heat-shock-induced translation inhibition is sufficient to recruit new mRNAs to germ granules. First, the 90% of mRNAs that are enriched in foci in the smFISH survey exhibited lower ribosome coverage on average than the 10% that were not enriched in foci (false positives). Second, quantitative smFISH on 12 transcripts revealed an anti-correlation between ribosome coverage and the percentage of molecules in germ granules. Third, relative enrichment in granules was reduced upon translational activation for four transcripts translated in P_4_. Finally, RNAi screening of transcripts enriched in granules and PGCs identified only a small subset that contribute to germ cell fate, ruling out a possible systematic preference for germ cell fate regulators. Together, these observations suggest that MEG-3 recruits to germ granules translationally repressed ribonucleoprotein complexes indiscriminately and in a reversible manner. It is unlikely that the 441 granule-enriched transcripts analyzed here represent the complete germ granule transcriptome as low-abundance, low-translation transcripts that were below the cut-off of the MEG-3 iCLIP were detected in germ granules when examined by smFISH (e.g. *lsl-1* and *xnd-1*; this study and Lee et al., 2020).

In *Drosophila*, mRNAs in polar granules de-mix into spatially separated ‘homotypic’ clusters containing multiple copies of the same mRNA species (Little et al., 2015; Niepielko et al., 2018; Trcek et al., 2015, 2020). We observed similar clusters for granule-enriched transcripts, with the most abundant and most granule-enriched transcripts forming the highest copy number clusters. These observations suggest that clustering is driven by phase separation of ribonucleoprotein complexes that exceed their critical concentration in the dense germ granule environment. The observation that different mRNAs tend to populate spatially distinct clusters is consistent with germ granules being agglomerates of compositionally distinct condensates (Cassani and Seydoux, 2022).

### Hypothesis: many mRNAs are recruited to germ granules to stabilize the protein-rich phases of germ granules

In addition to *Drosophila* and *C. elegans* germ granules, RNA clusters have been reported in the germ granules of zebrafish and in the giant condensates that form in arrested oocytes in *C. elegans* (Bontems et al., 2009; Cardona et al., 2023; Eno et al., 2019). In *C. elegans* oocytes, RNA clustering has been proposed to enhance translational repression (Cardona et al., 2023). In zebrafish, *nanos3* mRNA accumulates inside germ granules when translationally repressed early in development and on the granule surface coincident with translational activation (Westerich et al., 2023). Similarly, recent studies in *Drosophila* have reported that translation of *nanos* occurs at the granule surface (Chen et al., 2023 preprint; Ramat et al., 2023 preprint) where ribosomes accumulate (Mahowald, 1968). These studies imply that localization of mRNAs in granules contributes to their regulation.

We offer an alternative hypothesis: the translational status of an mRNA biases its propensity to phase separate with germ granule assembly proteins but is not determined by the granule environment. We suggest that clustering is a property of translationally repressed ribonucleoprotein complexes that is enhanced by non-specific RNA-binding proteins such as MEG-3 but is not essential for translational repression or RNA stability in germ plasm. Several observations support this model. First, we find that enrichment in germ granules and clustering does not predict whether an mRNA will be translated or turned over in the germline founder cell. Similarly, in *Drosophila*, certain germ granule transcripts are eventually degraded in PGCs whereas others are maintained (Hakes and Gavis, 2023). Furthermore, a survey of 11 *Drosophila* germ granule transcripts revealed variable timing in the onset of translation, which depended on 3′UTR sequences (Rangan et al., 2009). These observations suggest that RNA regulation is specific to each transcript and associated protein regulators, not the granule environment. Second, our analyses of *meg-3 meg-4* embryos indicate that RNA regulation is maintained even when the majority of RNA molecules are no longer enriched in clusters. The translational status of most transcripts (as revealed by ribosome profiling) does not change in *meg-3 meg-4* embryos (Lee et al., 2020). Furthermore, mRNA translation (*grif-1*, *nos-2*, *xnd-1* and *lsl-1*), mRNA degradation (*uba-1*, *oma-1*) and mRNA maintenance (*cgh-1*, *spn-4*) proceeds as in wild type in the germline founder cell of *meg-3 meg-4* embryos (this study; Cassani and Seydoux, 2022; Lee et al., 2020; Parker et al., 2020). Finally, two well-translated transcripts, although not enriched, were not excluded from germ granules, suggesting that the granule environment is compatible with translation, as recently demonstrated for stress granules (Mateju et al., 2020). We conclude that recruitment of mRNA molecules into clusters in germ granules is not essential for regulation of mRNA translation or stability.

If so, why are mRNAs recruited to germ granules? RNA enhances MEG-3 condensation *in vitro* (Lee et al., 2020) and the RNA-binding domain of MEG-3 enhances its condensation and ability to stabilize P granules *in vivo* (Schmidt et al., 2021). With RNA, MEG-3 forms nanoscale clusters that adsorb to the surface of liquid P granules where they act as ‘Pickering agents’ to reduce surface tension and stabilize P granules in germ plasm for transmission to PGCs (Folkmann et al., 2021). Deletion of the MEG-3 RNA-binding domain leads to less robust MEG-3 condensation and smaller P granules that are not efficiently transmitted to P_4_ (Schmidt et al., 2021). These observations suggests that MEG-3 binds mRNAs to stimulate its own condensation and Pickering activity.

Factors required for germ granule assembly, such as Xvelo1/Bucky ball in vertebrates, Oskar in *Drosophila* and MEG-3 in *C. elegans* evolved independently during animal evolution, but all share the ability to bind RNA and form low-dynamic, RNA-rich assemblies that recruit more dynamic proteins (Kulkarni and Extavour, 2017). We suggest that sequence non-specific condensation of germ granule assembly proteins with mRNAs not engaged with ribosomes creates low-dynamic, RNA-rich scaffolds, which in turn support the coalescence of protein-rich condensates by providing RNA-binding sites or by acting as Pickering agents (Folkmann et al., 2021). Reconstitution experiments in tissue culture cells have confirmed that non-specific, RNA-rich clusters can stabilize protein-rich condensates by interfacial adsorption (Garcia-Jove Navarro et al., 2019). Consistent with a transient scaffolding role in germ plasm, in *Drosophila*, RNA is required to maintain the Argonaute Aubergine in germ granules in oocytes (Curnutte et al., 2023) and is mostly cleared from germ granules after germ cell precursors have cellularized in embryos (Mahowald, 1971). Similarly, 12 of 26 oosome-enriched transcripts in *Nasonia* are not maintained in PGCs (Quan et al., 2019). A protein-centric model whereby mRNAs are recruited to germ granules to serve as scaffolds for protein condensation provides an explanation for why the germ granule transcriptome appears to include so many transcripts that are not essential for germ cell fate specification.

### A minority of germ granule mRNAs code for germ cell fate determinants and use the granules to accumulate in germ plasm

A protein-centric model for germ granules does not exclude the possibility that some transcripts coding for germ cell fate determinants evolved properties to enhance their capture by germ granule proteins to elevate their concentration in germ plasm. Consistent with this hypothesis, cross referencing of the 441 Group I and Group II genes identified in this study with 81 transcripts reported to localize to polar granules in *Drosophila* and/or the oosome in *Nasonia* (Quan et al., 2019; Rangan et al., 2009) identified ten shared homology groups with high to moderate confidence orthologs, including several with known germline functions in *C. elegans* (Table S2; Materials and Methods). The only transcripts shared by all three species were *nanos* orthologs, but given that the lists are likely incomplete, the overlap could be greater. These observations suggest that a subset of genes with germline functions are under selection to maintain RNA-encoded features that enhance localization to germ granules.

Studies in *Drosophila* concluded that efficient localization to polar granules depends on 3′ UTR sequences that enhance RNA clustering (Eagle et al., 2018; Valentino et al., 2022). The clustering efficiency of the *nanos* 3′ UTR varies across *Drosophila* species and correlates positively with PGC number (Doyle et al., 2023). Similarly, we observed strong clustering and enrichment in the germline founder cell for the *nanos* homolog *nos-2* and its redundant partner, the TRIM32 homolog *grif-1*. These properties, however, were not shared by all transcripts coding for germ cell fate determinants: *xnd-1* and *lsl-1* were concentrated in germ granules, but these low-abundance transcripts did not cluster, or enrich efficiently in the germline founder cell, despite being translationally activated in that cell. *xnd-1* and *lsl-1* code for nuclear factors that are also expressed zygotically during post-embryonic development, in contrast to *nos-2* and *grif-1*, which code for strictly maternal proteins that function during embryogenesis (Lee et al., 2020; Mainpal et al., 2015; Rodriguez-Crespo et al., 2022; Subramaniam and Seydoux, 1999). One possibility, therefore, is that efficient clustering in germ granules is a property under selection for the subset of germ cell fate regulators that are early acting and dose sensitive. Even for those transcripts, however, clustering in germ granules only serves to boost levels in germ plasm and the germline founder cell and is not essential for translational regulation.

### Implications for the role of germ granules in germ cell fate specification

Our findings suggest that the primary function of germ granules is to localize to the nascent germline a few maternal mRNAs coding for dose-sensitive germ cell fate determinants and dozens of proteins stabilized in the granules by low-translation mRNAs**.** How do proteins in germ granules contribute to germ cell fate? P granules concentrate Argonautes and other nuage proteins required for the biogenesis of silencing small RNAs (Batista et al., 2008; Beshore et al., 2011; Claycomb et al., 2009; Gu et al., 2009). *meg-3 meg-4* mutants fail to transmit maternal nuage to PGCs and accumulate over generations imbalances in silencing small RNAs that eventually silence RNAi genes, making *meg-3 meg-4* mutants incapable of mounting an RNAi defense (Dodson and Kennedy, 2019; Lev et al., 2019; Ouyang et al., 2019). Another condensate type in the *C. elegans* germ plasm are germline P-bodies, which accumulate regulators of mRNA translation and decay (Cassani and Seydoux, 2022). Germline P bodies co-segregate with P granules and are stabilized partially by MEG-3 and MEG-4 and primarily by MEG-1 and MEG-2, related intrinsically disordered proteins that lack the motif for targeting to P granules (Cassani and Seydoux, 2022). In *meg-1 meg-2* mutants, germline P-bodies are not efficiently transmitted to P_4_, transcripts like *nos-2* and *grif-1* are not translated efficiently, and others are not degraded, even though these transcripts still enrich with MEG-3-positive P granules as in wild type (Cassani and Seydoux, 2022). *meg-1 meg-2* mutants are 100% maternal-effect sterile with PGCs activating the transcription of somatic genes (Cassani and Seydoux, 2022). Condensates similar in protein composition to P granules and germline P-bodies have been reported in the *Drosophila* germ plasm (Cassani and Seydoux, 2023; Eichler et al., 2020; Hakes and Gavis, 2023). Together, these observations suggest that a conserved function of germ granules is to concentrate in germ plasm Argonaute/sRNA complexes and other regulators of mRNA translation and decay that shape the maternal-to-zygotic transition in the nascent germline.

### Limitations of this study

In this study, we use the term germ granules to refer to all RNA-rich condensates in germ plasm (including P granules and germline P-bodies). We did not explore whether different mRNAs localize to different condensate sub-types, as shown recently in *Drosophila* (Hakes and Gavis, 2023). Our conclusion that mRNAs do not need to cluster in germ granules for regulation is based on our analyses of eight transcripts in *meg-3 meg-4* embryos, which exhibited normal regulation despite most (>90%) mRNA molecules no longer residing in clusters. The few clusters that persist in *meg-3 meg-4* embryos could in principle contribute to RNA regulation, but only if transient interactions with clusters were sufficient for regulation. *meg-3 meg-4* embryos are ∼30% sterile (Wang et al., 2014). We speculate that this defect is a consequence of the failure to concentrate in the germline founder cell *nos-2*, *grif-1*, and possibly other transcripts coding for dose-sensitive germ cell fate regulators. We have not tested, however, whether artificially increasing the concentration of these mRNAs suppresses the *meg-3 meg-4* sterility phenotype. We cannot exclude, therefore, the possibility of another role for mRNA condensation or condensation-unrelated roles for MEG-3 and MEG-4.

## MATERIALS AND METHODS

### Worm handling and genome engineering

*C. elegans* were cultured at 20°C according to standard methods (Brenner, 1974). Genome editing was performed using CRISPR/Cas9 as described by Paix et al. (2017). Strains and oligos used in this study are listed in Table S1. A first *grif-1* deletion allele was generated in the N2 background to generate the single mutant *grif-1(ax4524)* using two guide RNAs flanking coding exons. A second, molecularly identical, deletion allele was generated in the strain DG3226 to generate the double mutant *grif-1(ax4533) deps-1(bn124)*. *grif-1* deletions were confirmed by Sanger sequencing. A deletion of the *cash-1* locus was generated using two guide RNAs flanking *cash-1* coding exons and was found to be zygotic lethal and was not maintained.

### Sterility counts

To determine the percentage of sterile worms in a population, 10-20 young adults were placed on standard NNGM plates (IPM Scientific) to lay eggs overnight. Adults were removed and embryos were left to develop into adults. One-hundred adult hermaphrodites were scored for empty gonads (sterile) by visual inspection under a low-magnification dissecting microscope (Zeiss Stemi 2000). For assessment of *grif-1 deps-1*; *nos-2* sterility, young adults were singled out onto standard NNGM plates, and all of their progeny were counted. Only worms with at least 30 progeny were reported.

### RNAi screen

RNAi knockdown experiments were performed by feeding (Timmons and Fire, 1998). RNAi plasmids were obtained from the Ahringer libraries and sequence verified or cloned from *C. elegans* cDNA and inserted into the L4440 RNAi vector. RNAi feeding vectors were transformed into HT115 bacteria, grown at 37°C in lysogeny broth (LB, Apex Bioresearch Products)+ampicillin (100 µg/ml, Sigma Aldrich) media for 5-7 h, induced with 1 mM IPTG (Gold Biotechnology) for 30 min, and plated on RNAi plates (50 µg/ml carbenicillin+1 mM IPTG, IPM Scientific). Five adults were bleached onto RNAi plates to isolate embryos, which were grown on RNAi bacteria to adulthood before transferring to standard NNGM plates and allowed to self to generate F1 progeny. For each RNAi treatment, 100 adult F1 progeny were scored for empty uteri (sterile).

### smFISH probes

smFISH probes used in this study were designed using Biosearch Technologies Stellaris Probe Designer and ordered from the same with Quasar570 or Quasar670 probes or ordered as DNA oligos from IDT and labeled in the lab with either Cy3 or Cy5-NHS ester using a protocol adapted from Gaspar et al. (2017). Oligos were labeled with amino-11-ddUTP at the 3′-end using terminal deoxynucleotidyl transferase, purified on a Spin-X centrifuge column loaded with Bio Gel P-4 Beads prior to reacting with Cy3 or Cy5-NHS esters. Non-conjugated dyes were removed in a second round of purification on a Spin-X centrifuge column. Labeling efficiency and yield were determined using a Nanodrop spectrophotometer (Thermo Fisher Scientific NanoDrop One)

### smFISH

For smFISH, embryos were extruded from adult animals and subjected to freeze-cracking on 0.01% poly-lysine coated slides, followed by fixation in −20°C methanol overnight. Slides were washed once in 1:1 methanol:PBSTw, five times in PBSTw (1× PBS+0.1% Tween-20), and fixed in 4% paraformaldehyde in PBS for 1 h at room temperature. Fixed slides were washed four times in PBSTw, twice in 2× SSC, once in wash buffer (10% formamide, 2× SSC), and blocked in hybridization buffer (10% formamide, 2× SSC, 200 µg/ml bovine serum albumin, 2 mM Ribonucleoside Vanadyl Complex, 0.2 mg/ml yeast total RNA, 10% dextran sulfate) for 30 min at 37°C in a humid chamber. Slides were incubated with probes in hybridization buffer at 37°C overnight, washed twice in wash buffer at 37°C for 30 min, twice in 2× SSC, once in PBSTw, and twice in PBS before mounting in ProLong Glass Antifade Mountant with NucBlue Stain (Invitrogen) and cured overnight.

Images of probe-hybridized embryos were collected across seven stages (two-cell, four-cell, eight-cell, 28-cell, ∼40-cell, ∼60-cell, ∼100-cell). Two-cell, four-cell, eight-cell and 28-cell embryos were scored manually for granules visible in P_1_, P_2_, P_3_ and P_4_ blastomeres (P blastomeres are readily identified at these stages based on their position in the embryo). Transcripts positive for granules were classified as Group I or II, and transcripts negative for granules were classified as Group III.

The distinction between Group I and Group II transcripts was made by examining ∼40-cell, ∼60-cell and ∼100-cell stage embryos. Transcripts that showed consistent enrichment in one cell (40-cell and 60-cell stages) and two cells (100-cell stage) across these stages were classified as Group I. Because the P lineage is the only lineage that progresses from one to two cells in this developmental period and area of the embryo, this pattern is consistent with transcript maintenance in the P lineage to the PGC stage. Transcripts that did not exhibit this pattern were classified as Group II (transcripts not enriched in PGCs). We note that five Group III transcripts (*vdac-1*, *gld-1*, *F22D6.2*, *dlc-1* and *act-2*) exhibited a pattern consistent with maintenance to the PGC stage, despite no apparent enrichment in granules.

Representative images from the *in situ* hybridization screen are accessible from the Dryad Digital Repository (Scholl, 2024): dryad.02v6wwq98. *In situ* hybridization results are tabulated in Table S3, Fig. 1 and Fig. S1B,D,E.

### Spinning disk confocal microscopy

Microscopy was performed either using a Zeiss Axio Observer equipped with a CSU-W1 SoRA spinning disk scan head (Yokogawa) and an iXon Life 888 EMCCD camera (Andor) using a 40× objective with a 4× relay lens (Yokogawa) (Fig. 1; Fig. S2A; Fig. 3B,C) or a Zeiss Axio Imager Z1 equipped with a CSU-W1 SoRA spinning disk scan head (Yokogawa) and an ORCA-Fusion BT digital CMOS camera (Hamamatsu) using a 63× objective with a 2.8× relay lens (Yokogawa) (Figs 3D,E, 4-6; Fig. S3). Images were taken using SlideBook software (Intelligent Imaging Innovations). For Fig. S3A, embryos were imaged live and brightfield illumination was used to determine the approximate cell stage.

### Quantification of RNA enrichment in P_4_

Images of *z*-slices centered on the nucleus of P_1_ or P_4_ blastomeres were obtained using a Zeiss Axio Observer as above. To calculate enrichment in P_4_ relative to P_1_, the raw integrated density of each *z*-slice was measured using Fiji. Background signal was determined as the raw integrated density in the nucleus. The concentration for each P cell [P] was computed as (intensity/area of cell) – (intensity/area of nucleus). For the data shown in Fig. 3, enrichment for each P_4_ cell was calculated as [P_4_]/average [P_1_] (calculated from three P_1_ blastomeres).

### Airlocalize analyses

High-resolution smFISH images were obtained using a Zeiss Axio Imager Z1 as above and analyzed using Airlocalize (Lionnet et al., 2011; Trcek et al., 2015). Entire *z*-stack *in situ* hybridization images were processed in Fiji by applying a background subtraction of 50 pixels. All images from a single experiment were manually thresholded together. To identify intensities corresponding to single mRNA molecules, images of AB and EMS blastomeres were used. PSF Width and Detection Threshold were initially set interactively, and five foci were selected and fitted with a Gaussian curve to set the PSF. The threshold was adjusted until all foci were detected by the program. The same PSF and threshold value was used on all images of the same channel taken on the same day using batch mode. The median of the Integrated Intensity (outputted from Airlocalize) for all AB and EMS images for a given transcript was assigned as the intensity of a single mRNA molecule for that transcript. That intensity value was used to bin foci corresponding to two, three or more molecules (Fig. S4). For analyses shown in Figs 4B and 6A,D, we analyzed an average of 947 molecules per RNA and stage in a minimum of four P blastomeres. Image brightness was enhanced for the panels shown in Fig. 4A for display purposes.

To identify MEG-3 granules, entire *z*-stack images were processed in Fiji by applying a background subtraction of 50 pixels. A Gaussian blur filter of 1 sigma was applied. Images were automatically thresholded using the MaxEntropy settings, and adjustments were made to the threshold if granules were not detected. To separate out individual granules, the Fiji watershed method was applied. Granules were segmented using the 3D ROI Manager, and regions of interest (ROIs) were manually checked to identify those that needed to be split further. Once all ROIs were selected, 3D measurements were collected. To determine colocalization of RNA clusters in MEG-3 granules, the *xyz* coordinates of clusters (cluster center) outputted from Airlocalize were used (with the *x* and *y* coordinates flipped in Fiji). The *xyz* boundaries of MEG-3 granules were calculated as a cube using the 3D measurements from Fiji. The width and length of a granule were estimated as (minor radius+2)*2 (2 pixels were added to allow room for error), and the height was estimated as (major radius)*2 (granules seemed to be largest in height). An RNA cluster was considered to be in a granule if its *xyz* coordinates (cluster center) were within the boundaries of the calculated cube. Clusters from two different RNAs were considered colocalized in MEG-3 granules if within all distance thresholds (2 pixels in *x* and *y*, 2 slices in *z*).

To determine the percentage of cytoplasm occupied by MEG-3 or EDC-3 granules, 3D measurements of the granules, P cell, and nucleus were collected. The cytoplasmic volume was calculated as the P cell volume minus the nucleus volume. The percentage of cytoplasm occupied by granules was calculated as the total volume of MEG-3 or EDC-3 granules divided by the cytoplasmic volume.

Python codes used for processing Airlocalize data and colocalization analysis have been deposited in Github: https://github.com/yliu380/AirLocalize_Data_Process.

### Orthology analysis

To identify evolutionarily conserved germ granule transcripts, we identified the *C. elegans* orthologs of 58 *Drosophila* transcripts identified as germ plasm-enriched by Rangan et al. (2009) and 26 *Nasonia* transcripts identified as oosome-enriched by Quan et al. (2019). The *Nasonia* genes were converted to *Drosophila* orthologs as listed by Quan et al. (2019) or by BLASTing against Fly Base (Nv-igf). Eight *Nasonia* genes with no *Drosophila* orthologs were analyzed manually using OrthoDB to identify *C. elegans* orthologs (LOC100123551). *Drosophila* genes/orthologs were batch processed in DIOPT (https://www.flyrnai.org/diopt) to identify high-, moderate- and low-confidence *C. elegans* orthologs, which were cross-referenced to the Group I and II lists. These analyses yielded 13 Group I or II genes with high- or moderate-confidence orthologs in *Drosophila* or *Nasonia* and 11 with low-confidence orthologs (Table S2).

## Supplementary Material

10.1242/develop.202575_sup1 Supplementary information

Table S1. List of strains used in this study.

Table S2. Conserved germ granule transcripts identified by comparing *C. elegans* Group I and II RNAs (this study), *Drosophila* germ plasm-enriched transcripts (Rangan et al., 2009), and *Nasonia* oosome-enriched transcripts (Quan et al., 2019). See Methods for identification of *Nasonia* orthologs. Full list of transcripts (Table S2 transcripts) used for DIOPT analysis (Table S2 DIOPT analysis) listed in separate tabs.

Table S3. Complete description of *in situ* results and raw values used to generate graphs.
