# Supplementary material for: Caenorhabditis elegans germ granules accumulate hundreds of low translation mRNAs with no systematic preference for germ cell fate regulators
Source: Development. 2024 Jul 10;151(13):dev202575. doi: 10.1242/dev.202575 (PMC11266749; doi:10.1242/dev.202575)
Supplement: Supplementary information [file develop-151-202575-s1.pdf]

Figure S1

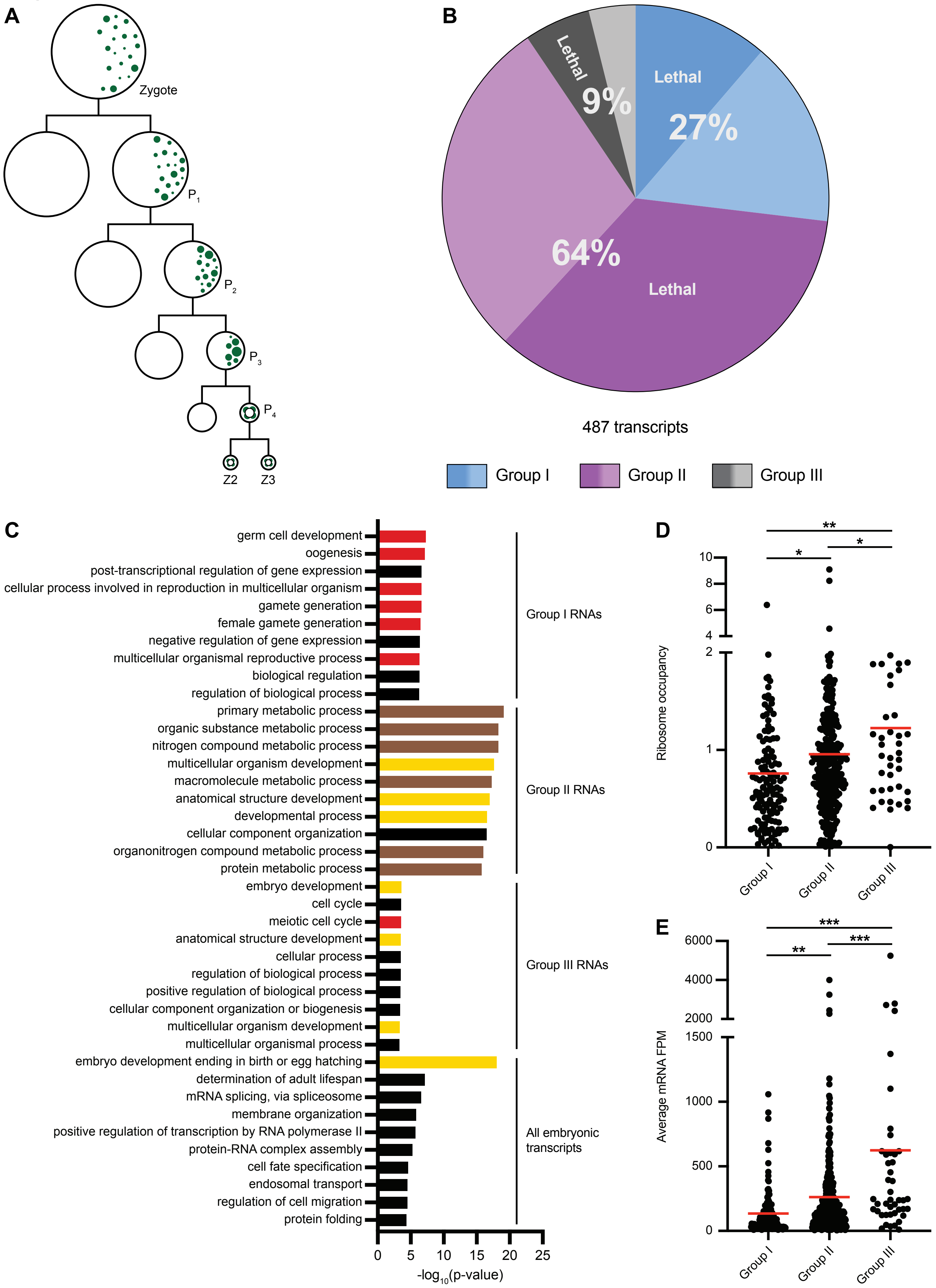

Development • Supplementary information

Fig. S1. Characterization of MEG-3-bound transcripts

(A) Lineage showing the asymmetric divisions that divide the zygote into somatic blastomeres and P blastomeres (P_1-P_4) that contain germ granules (green dots). Divisions of somatic blastomeres are not shown.

(B) Pie chart showing distribution of the 487 transcripts into Groups I, II, and III. Darker colors represent genes whose knock-down led to embryonic lethality in wild-type or *rrf-3* (enhanced RNAi) hermaphrodites as reported in WormBase version WS289 (Davis et al., 2022).

(C) Graph showing the gene ontology (GO) terms significantly enriched (*P*-value < 0.01) in Groups I, II, and III (Figure 1) and embryonic transcripts expressed at levels comparable to MEG-3-bound RNAs (Lee et al., 2020). Only the top 10 GO terms are shown. Similar GO terms are color-coded in red (germline), brown (metabolism), and yellow (development).

(D,E) Average ribosome occupancy or mRNA abundance (FPM, Y-axis; Lee et al., 2020) for each Group (X-axis). Each dot represents a transcript. ***$P \leq 0.001$, **$P \leq 0.001$, *$P \leq 0.05$ (unpaired *t*-test).

Development • Supplementary information

Figure S2

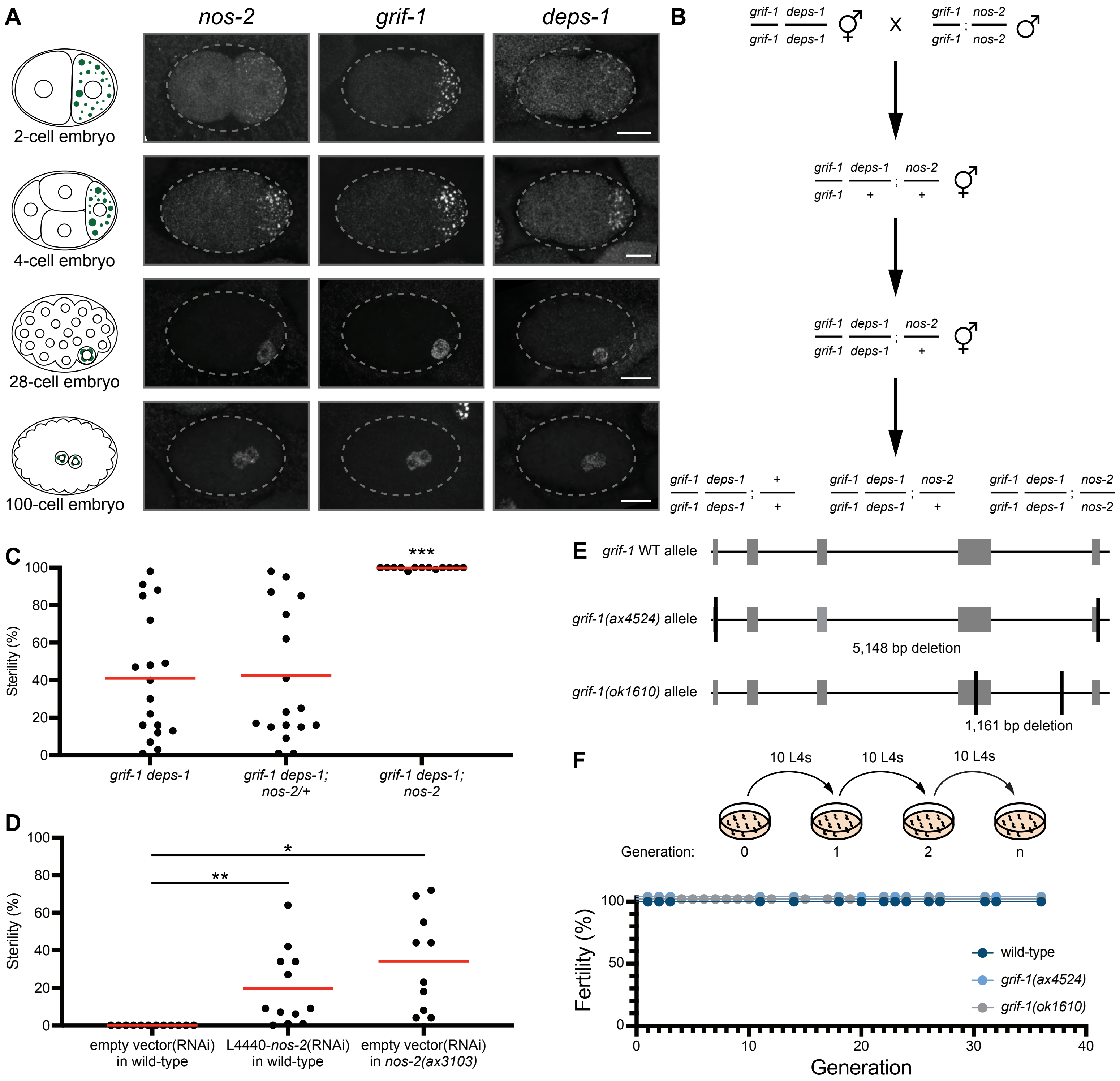

Development • Supplementary information

Fig. S2. *grif-1*, *deps-1* and *nos-2* are required redundantly for germ cell specification

(A) Representative photomicrographs of wild-type embryos of indicated stage and hybridized to gene-specific smFISH probes as indicated in top row. Dashed lines indicate embryo boundary. Images are maximum projections. The *nos-2* and *grif-1* panels show the same 4-cell embryo hybridized to the two probes. Scale bars: 10 μm.

(B) Mating scheme used to generate genotypes shown in Fig. S2C. Animals were genotyped by PCR after selfing.

(C) Graph showing the percent sterility (Y-axis) of the genotypes listed (X-axis). Each dot (biological replicate) corresponds to a brood (>30 self-progeny) derived from a single mother of the indicated genotype as generated by the scheme shown in B. Red lines indicate the mean. ***$P \leq 0.0001$ (unpaired *t*-test compared to *grif-1 deps-1* siblings). The data for the triple mutant are also shown in Fig. 2B.

(D) Graph showing the percent sterility (Y-axis) of the conditions listed (X-axis). Each dot (biological replicate) corresponds to a brood (up to 100 self-progeny counted) from 10-20 mothers. Red lines indicate the mean. **$P \leq 0.001$, *$P \leq 0.01$ (unpaired *t*-test).

(E) Schematic showing exons (rectangles) and introns (lines) at the *grif-1* locus. Vertical lines indicate boundaries of the deletions in two *grif-1* alleles.

(F) Schematic showing the protocol for measuring sterility over generations. Graph below shows the percent fertility (Y-axis) of each generation (X-axis) for wild-type (dark blue), *grif-1(ax4524)* (light blue), or *grif-1(ok1610)* (gray). Data show averages from two experimental replicates for each allele. Unlike Oyewale and Eckmann, 2022, we noted no significant sterility over 30+ generations in either *grif-1* allele.

Development • Supplementary information

Figures S3

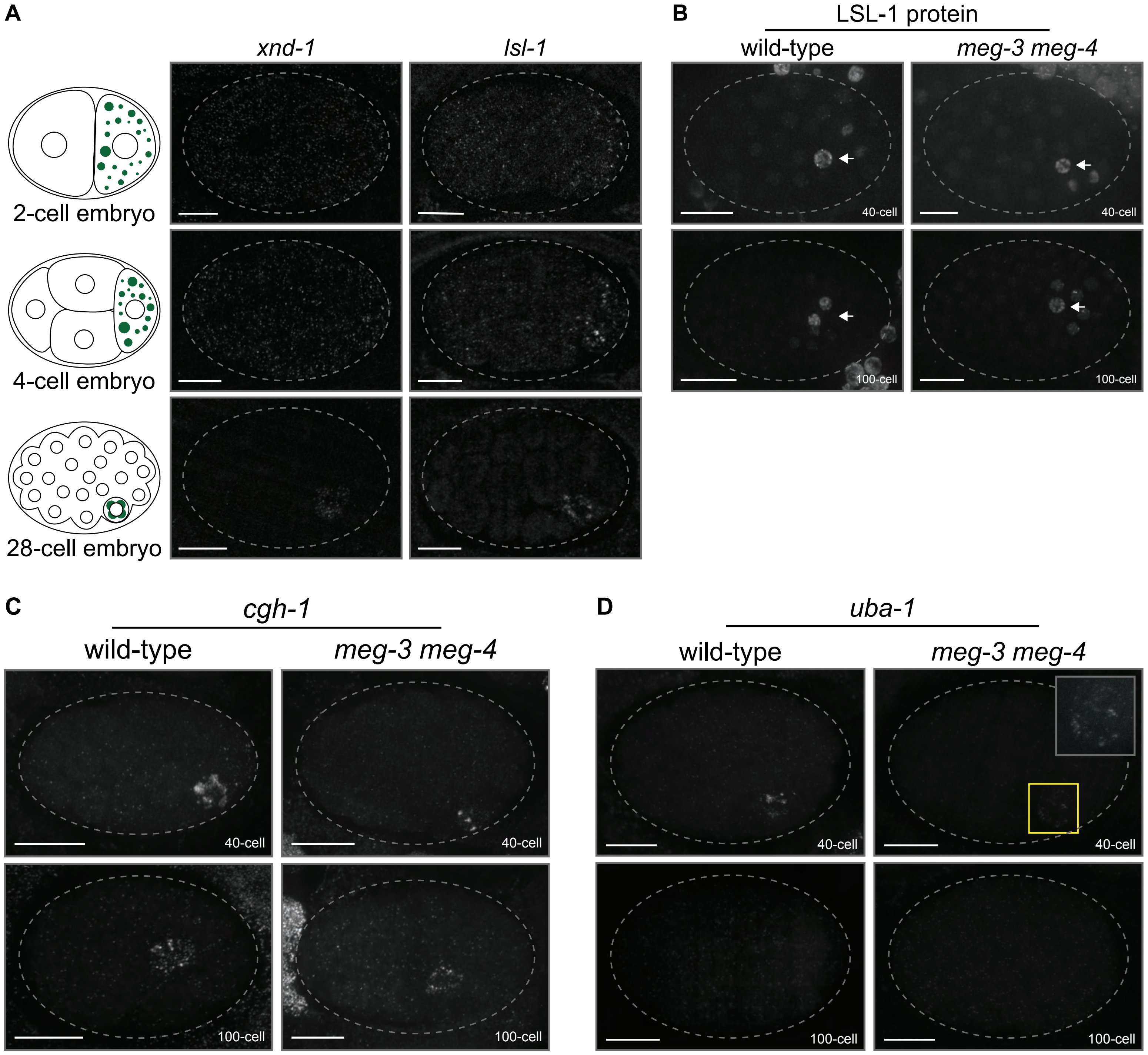

Fig. S3. MEG-3 is not required for RNA regulation

(A) Representative photomicrographs of wild-type embryos hybridized to *xnd-1* or *lsl-1* smFISH probes. Images are maximum projections.

(B) Embryos expressing a LSL-1::GFP fusion engineered at the endogenous locus (Rodriguez-Crespo et al., 2022). LSL-1::GFP is detected in the nuclei of P_4 and descendants (arrow) and at lower level in the P_4 sister blastomere D and its descendants in both wild-type and *meg-3 meg-4* mutants.

(C-D) Representative photomicrographs of embryos of indicated genotype and stage hybridized to gene-specific smFISH probes, showing *meg-3 meg-4*-independent maintenance (C) or turnover (D) of transcripts from P_4 (40-cell) to Z2 and Z3 (100-cell). Images are maximum projections. Scale bars: 10 μm.

Development • Supplementary information

Figure S4

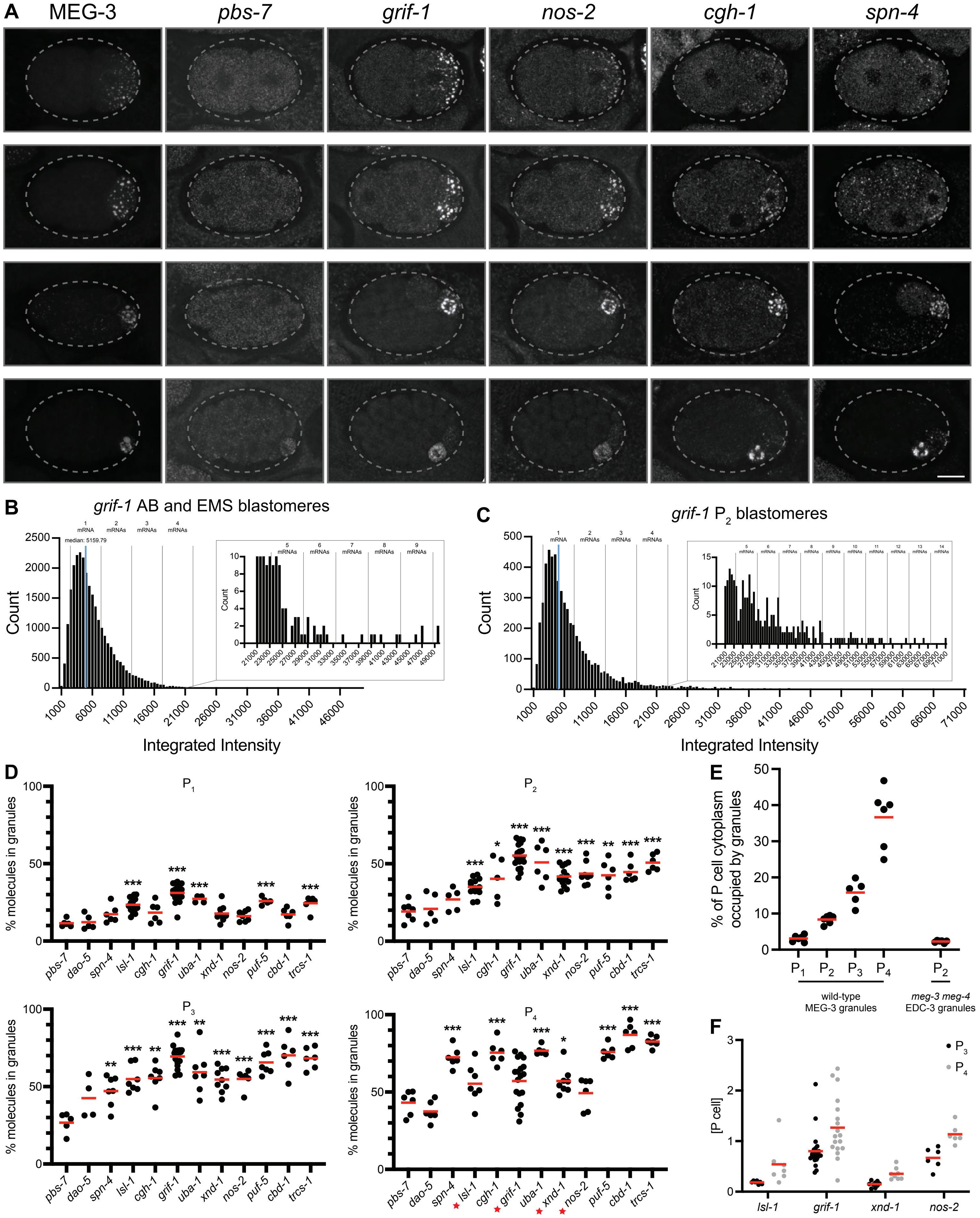

Development • Supplementary information

Fig. S4. RNA enrichment in germ granules using Airlocalize

(A) Representative photomicrographs of embryos of indicated stage and hybridized to gene-specific smFISH probes as indicated in top row. Embryos are expressing MEG-3::GFP. Images are maximum projections. Embryos were co-stained for *grif-1* and *nos-2* or for *cgh-1* and *spn-4*. MEG-3::GFP and *pbs*-images are the same embryo. Scale bar: 10 μm.

(B) Graph showing the number of smFISH dots (Y-axis) with a given integrated intensity (X-axis) in Airlocalize-processed FISH images of *grif-1* RNA in AB and EMS somatic blastomeres. The median (blue line) was used to set thresholds for intensities corresponding to 1, 2, 3, or 4 or more RNAs. Intensities corresponding to 4 or more mRNAs are referred to as "clusters" in the text. Inset shows an expanded view of the graph.

(C) Same as above but for P_2 blastomeres. Note the expanded X axis.

(D) Graphs showing the percent of molecules in germ granules (Y-axis) for the RNAs listed (X-axis) in P blastomeres. Each dot corresponds to one P blastomere. Red lines indicate the mean and were used to generate Fig. 4B. ***$P \leq 0.0001$, **$P \leq 0.001$, *$P \leq 0.01$ (unpaired *t*-test compared to *pbs-7*).

(E) Graph showing the percent of cytoplasmic volume occupied by granules (Y-axis) in P blastomeres (X-axis). Each dot corresponds to one P blastomere. Red lines indicate the mean. Consistent with germ granules segregating into successively smaller P blastomeres (Fig. S1), MEG-3::GFP granules occupy an increasingly higher percent of cytoplasmic volume with each P blastomere. The last bar shows the percent of cytoplasmic volume occupied by granules marked by EDC-3 in *meg-3 meg-4* P2 blastomeres. The MEG-3 and EDC-3 average values in P2 blastomeres are shown as dotted lines in the bar graphs shown in Fig. 6E,F.

(F) Graph showing P_3 and P_4 concentrations (Y-axis) for the four transcripts that are translationally activated in P_4 (X-axis). Total number of molecules detected in the cell divided by average cell volume of P_3 and P_4 from Fig. S4E was used to estimate concentration. Values increase from P_3 to P_4, suggesting that the reduction in granule association in P_4 (Fig. S4D) is not due to degradation.

Development • Supplementary information

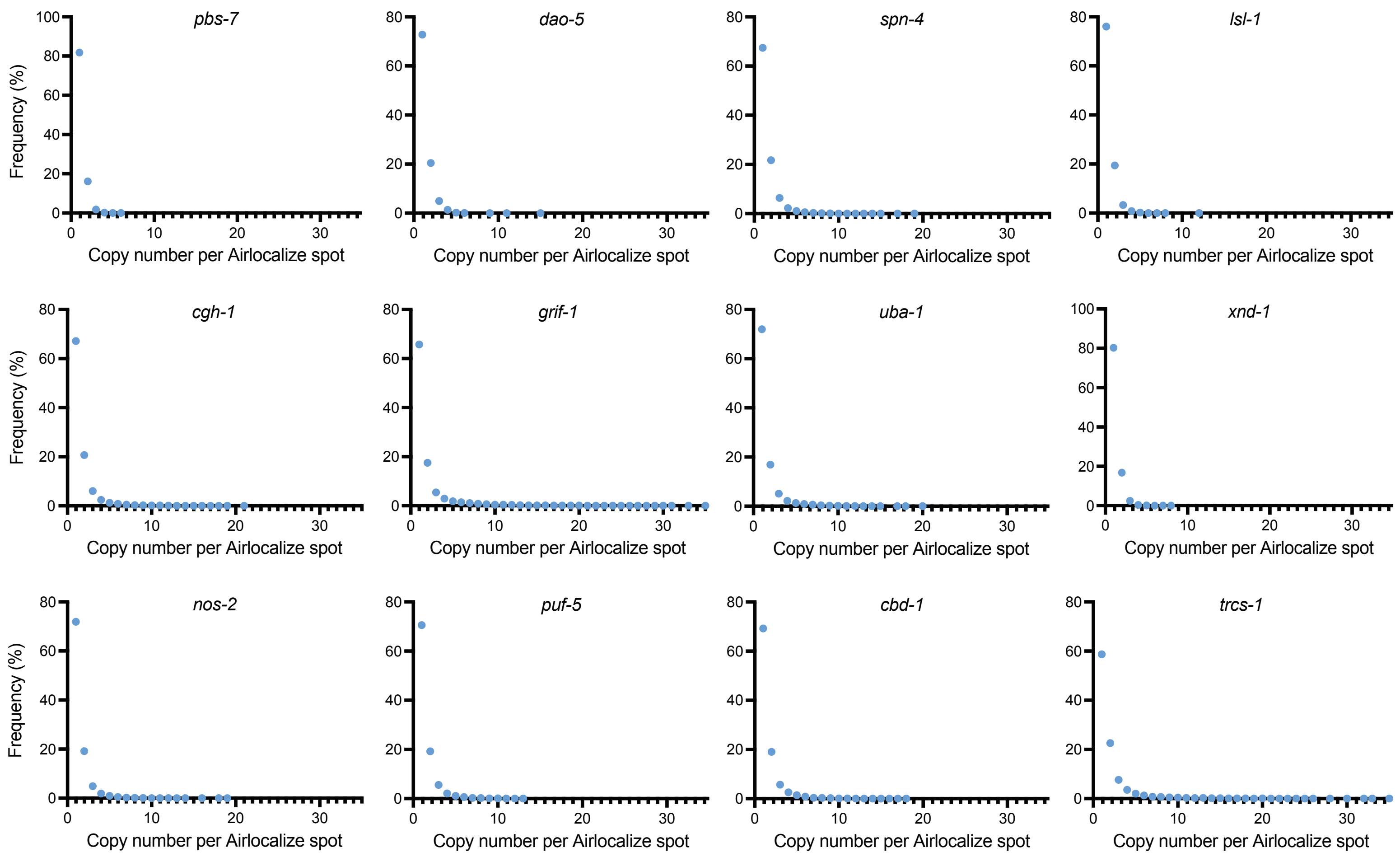

Fig. S5. Airlocalize spot distribution of RNA transcripts

Graphs showing the frequency (Y-axis) of clusters containing a specific number of RNA molecules per Airlocalize spot (X-axis) combining data from P_1 through P_4 blastomeres.

Table S1. List of strains used in this study.

Available for download at

https://journals.biologists.com/dev/article-lookup/doi/10.1242/dev.202575#supplementary-data

Table S2. Conserved germ granule transcripts identified by comparing *C. elegans* Group I and II RNAs (this study), *Drosophila* germ plasm-enriched transcripts (Rangan et al., 2009), and *Nasonia* oosome-enriched transcripts (Quan et al., 2019). See Methods for identification of *Nasonia* orthologs. Full list of transcripts (Table S2 transcripts) used for DIOPT analysis (Table S2 DIOPT analysis) listed in separate tabs.

Available for download at
https://journals.biologists.com/dev/article-lookup/doi/10.1242/dev.202575#supplementary-data

Table S3. Complete description of *in situ* results and raw values used to generate graphs.

Available for download at

https://journals.biologists.com/dev/article-lookup/doi/10.1242/dev.202575#supplementary-data

Development • Supplementary information
